# Comparison of Cox’s Regression Model and Parametric Models in Evaluating the Prognostic Factors for Survival after Liver Transplantation in Shiraz during 2000–2012

**Published:** 2015-08-01

**Authors:** R. Adelian, J. Jamali, N. Zare, S. M. T. Ayatollahi, G. R. Pooladfar, N. Roustaei

**Affiliations:** 1 Department of Pediatrics, Faculty of Medicine, Shiraz University of Medical Sciences, Shiraz, Iran; 2 *Department of Biostatistics, Faculty of Medicine, Shiraz University of Medical Sciences, Shiraz, Iran*

**Keywords:** Survival analysis, Adult, Pediatrics, Liver transplantation

## Abstract

**Background:**

Identification of the prognostic factors for survival in patients with liver transplantation is challengeable. Various methods of survival analysis have provided different, sometimes contradictory, results from the same data.

**Objective:**

To compare Cox’s regression model with parametric models for determining the independent factors for predicting adults’ and pediatrics’ survival after liver transplantation.

**Method:**

This study was conducted on 183 pediatric patients and 346 adults underwent liver transplantation in Namazi Hospital, Shiraz, southern Iran. The study population included all patients undergoing liver transplantation from 2000 to 2012. The prognostic factors sex, age, Child class, initial diagnosis of the liver disease, PELD/MELD score, and pre-operative laboratory markers were selected for survival analysis.

**Result:**

Among 529 patients, 346 (64.5%) were adult and 183 (34.6%) were pediatric cases. Overall, the lognormal distribution was the best-fitting model for adult and pediatric patients. Age in adults (HR=1.16, p<0.05) and weight (HR=2.68, p<0.01) and Child class B (HR=2.12, p<0.05) in pediatric patients were the most important factors for prediction of survival after liver transplantation. Adult patients younger than the mean age and pediatric patients weighing above the mean and Child class A (compared to those with classes B or C) had better survival.

**Conclusion:**

Parametric regression model is a good alternative for the Cox’s regression model.

## INTRODUCTION

Liver transplantation is the treatment of choice for end-stage liver disease (ESLD) and acute fulminant hepatitis [1,2]. In most cases, a liver transplant from a brain-dead person is performed with the consent of their relatives. Based on statistics reported on the Donation and Transplantation Institute and Transplant Procurement Management about International Registry in Organ Donation and Transplantation (IRODaT), the percentage of people who donated their organs after death in 2012 in Spain, USA, UK, Iran, Turkey, and Japan were 35.1, 25.6, 18.3, 6.9, 4.5, and 0.9 per million population (pmp), respectively [3]. Liver transplantation is used routinely worldwide. The success rate of liver transplantation and patient survival are on the rise due to advancements in medicine and immunosuppressive agents [4, 5]. Children comprise 15% to 20% of patients who are in waiting lists for a liver transplant [6]. Iran, according to IRODaT ranking, ranked 7^th^ based on living donors worldwide (19.7 and 0.4 pmp in kidney and liver transplantation, respectively) [3]. In Iran, liver transplantation has been performed in Namazi Hospital, Shiraz, southern Iran, since 1999. Patients come to this center from all over the country [7, 8]. 

Several studies have been conducted to identify the prognostic factors of patients’ survival after transplantation. In survival data modeling, one of the main objectives is to determine effective factors on survival time. A popular regression model for the analysis of survival data is the Cox’s regression model. The Cox’s regression model is a semi-parametric model making fewer assumptions than typical parametric methods and therefore it is the most practical and well-known statistical model to investigate the relationship between predictors and the time-to-event through the hazard function [9, 10]. In this model, there was no need for the researcher to assume a particular survival distribution for the data [11]. The only assumption made in the model is about the proportional hazards and this is why it is also called Cox proportional hazards regression [12, 13].

Unlike the Cox’s regression model that does not specify the distribution function of hazard function, there are several parametric models such as Weibull, exponential, log-normal, and log-logistic models where hazard function has to be specified [5, 14]. Studies have indicated that under certain circumstances, such as strong effect or strong time trend in covariates or follow-up depending on covariates, parametric models are good alternatives to the Cox’s regression model [11, 12, 14]. If the parametric models better fit the data, a more precise estimation of parameters would be achieved [15]. Maximum likelihood (ML) is used for estimation of parameters in survival parametric models, while Cox’s regression model is used for partial likelihood [16].

In this study, we compared the parametric methods (Weibull, exponential, log-normal, and log-logistic) and Cox’s regression model to determine the independent factors for predicting patients’ survival after liver transplantation in Namazi Hospital, Shiraz, southern Iran.

## PATIENTS AND METHODS

In this study we reviewed the database of the Liver Transplant Unit, Namazi Hospital, Shiraz, southern Iran. The included records of all (n=529) patients who had undergone liver transplantation between were 2002 and 2012. Various studies showed different independent predictors for adults and pediatrics patients’ survival. Therefore, the dataset was divided into two groups—pediatric patients (age <18 years) and adults (age ≥18 years).

Prognostic factors such as sex, age, Child class, initial diagnosis of the liver disease, Model for ESLD/Pediatric ESLD (PELD/MELD) score and pre-operative laboratory markers (serum albumin, international normalization ratio [INR], total bilirubin, direct bilirubin, blood urea nitrogen (BUN), creatinine, and white blood cell count) were recorded for patients. The PELD/MELD score is based on a patient’s risk for death in wait list for receiving a liver transplant. The score is a predictor for the transplant outcome. For the calculation of MELD score (for those aged >12 years) serum creatinine, total bilirubin and INR are considered [17]. Parameters used in the calculation of PELD score (for those aged <12 years) include age, serum albumin, total billirubin, INR and whether the patient has growth retardation [18].

Child-Pugh score (for all age ranges) is used to assess the severity of the liver disease. Scores 5–6 is considered “Child A,” 7–9 “Child B,” and scores ≥10 is considered “Child C.”

Proportional hazard model is a class of survival models that assesses the relationship between one or more covariates with time. One of the advantages of this model is it does not require strong assumptions on the distribution of data [19]. This model consists of two parts: the underlying hazard function, often denoted as *h*_0_*(t)*, describing how the risk of the event per time unit changes over time at baseline levels of covariates; and the effect parameters describing how the hazard varies in response to explanatory covariates (*X* is a vector of explanatory covariates and *β* is a vector of unknown regression parameters). The hazard function in Cox’s regression model is given by [20]:

Weibull distribution is one of the flexible parametric models to study life time data used widely in medicine. The hazard function of this distribution can be increasing, decreasing, or constant. The simplest one-parameter model is an exponential distribution. This distribution is a special case of the Weibull distribution. The hazard function is constant when the survival time is exponentially distributed. 

Log-logistic is another alternative model for the Weibull distribution. The hazard rate in this distribution is hump-shaped (it first increases and then decreases). Log-normal is another distribution which is widely used in medical sciences. The shape of the hazard rate log-normal is the same as the log-logistic. In many cases, regression models based on a log-normal distribution is very close to regression models based on the log-logistic distribution. We also considered this model because the baseline hazard has the value of 0 for *t*=0 and is hump-shaped [15,20].

AIC Criteria

The best model was selected based on the lowest Akaike information criterion (AIC) value. For parametric models discussed, the AIC is given by:

Where *p* is the number of parameters in the model; *k=1* for the exponential model; *k*=2 for the Weibull, log-logistic, and log-normal models [15].

Using SAS^®^ ver 9.3, Cox’s regression and parametric models were fitted to determine the independent prognostic factors of patients’ survival.

## RESULT

Among 529 patients who underwent living-related-donor liver transplantation, 346 (64.5%) were adult and 183 (34.6%) were pediatric patients. In adult group, there were 70.2% males; it was 54.4% in pediatric cases. In adult and pediatric patients 79.8%, 80.9% have survived, and 20.2%, 19.1% died after the transplantation, respectively.

The mean±SD age and weight in adults was 39.16±12.42 years and 67.47±13.17 kg, respectively. The values in the children were 7.93±5.50 years and 24.02±16.7 kg, respectively.

The mean±SD MELD/PELD score in adult and pediatric patients was 20.69±5.55 and 17.79±7.04, respectively. The most common initial diagnosis of the liver disease in patients was hepatitis (n=192, 55.5%) in adults and cholestatic disorders (n=95, 51.9%) in children (Table 1).

**Table 1:** Indications for liver transplantation in 183 children and 346 adults in Namazi Hospital

Diagnosis	Adult n (%)	Pediatric n (%)
Hepatitis	192 (55.5)	29 (15.8)
Cholestatic	117 (33.8)	95 (51.9)
Congenital metabolic disorder	22 (6.4)	56 (30.6)
Others	15 (4.3)	3 (1.6)

More than half (53.2%) of adults and 53.6% of children had Child C class; 42.5% of adults and 53.6% of the pediatric patients were in Class B. The rate of complication after the liver transplantation in adults and children was 42.2% and 26.2%, respectively. The most common complication was rise in the liver enzymes in adults and neurological problems (convulsion, *etc*) in children. The laboratory findings in adults and pediatric patients who referred for liver transplantation are shown in Table 2.

**Table 2:** The laboratory data in adult and children patients who underwent liver transplantation. Data are presented as mean±SD (min–max).

	Adult	Pediatric
Total bilirubin (mg/dL)	6.17±7.75 (0–72)	10.23±12.20 (0–60)
Direct bilirubin (mg/dL)	2.29±3.17 (0–18)	3.08±4.25 (0–18)
INR	2.27±1.45 (1–20)	1.79±1.20 (1–8)
Albumin (g/dL)	3.33±0.65 (1–5)	3.97±0.82 (1–6)
BUN (mg/dL)	16.03 ±8.816 (3–74)	13.16±7.21 (3–51)
Creatinine (mg/dL)	0.94±0.36 (0–3)	0.62±0.41 (0–5)
White blood cell count (mm^3^)	5568.82±3334.44 (900–27300)	7418.42±4065.09 (1200–24700)

Cox’s Regression Model

To determine the independent predictors for patients’ survival after the liver transplantation, we studied a Cox’s regression model. A proportional hazard (PH) was investigated for each variable using *log(-log(s(t))* against *log(t)* plot. Correlation between ranking of individual failure times and the Schoenfeld residuals for a particular was considered. If the PH assumption is met, then the correlation should be near zero [20]. The Schoenfeld residual-based test showed that the PH assumption was held for all the factors (p>0.05 for all factors).

At first, each variable was separately entered in a Cox’s model; then, the variables that had a p<0.2 were entered into multiple Cox’s regression model for adults and pediatric cases, separately.

On the whole, the adult age, albumin, and the initial diagnosis of the liver disease were entered into multiple Cox’s regression model; age was identified as an independent predictor for adults’ survival (HR=2.10, p<0.01). For children, weight, albumin, and Child class were entered into the model; weight was found the independent predictor for pediatric patients’ survival (HR=4.29, p<0.01).

Parametric Models

The variables entered into multiple Cox’s regression model fitted the parametric models such as Weibull, exponential, log-logistic, and log- normal. All the parametric models were well fitted with respect to graphical methods. For assessing the exponential distribution, the plot of *–log(S(t))* against *t* should yield a straight line with no abscissa; for Weibull distribution, the plot of *log(logS(t))* against *log(t)*; for log-normal distribution, the plot of *Φ*^-1^*(1*-*s(t))* against *log(t),* where *Φ* is the cumulative distribution function (c.d.f); and for log-logistic, the plot of *log((1-s(t))/s(t))* against *log(t)* should be a straight line.

Among the models used, the one with the lowest AIC was identified as the best model. The log-normal model with the lowest AIC, among parametric models and Cox’s regression were the best model in adult and pediatric patients. According to results from log-normal model in adults, age (HR=1.46, p<0.05), and in pediatrics, weight (PH=2.68, p<0.01) and Child class B (HR=2.12, p<0.05) were the significant factors. AIC criteria for Cox’s regression model and HR parametric models are shown in Tables 3 and 4. 

**Table 3:** Parametric regression model and Cox’s regression model fitted to adult patients’ data

covariate	Log-normal		Weibull		Log-logistic		Exponential		Cox’s model	
	HR^†^	SE^‡^	HR	SE	HR	SE	HR	SE	HR	SE
Age										
>mean	1		1		1		1		1	
<mean	1.46*	0.80	2.10**	0.74	2.17**	0.76	2.45**	0.26	2.10**	0.26
Cause										
Metabolic disorder	1		1		1		1		1	
Hepatitis	1.80	2.25	3.39	2.82	3.57	2.72	3.31	1.02	3.36	1.02
Cholestatic	1.97	2.27	4.17	2.84	4.55	2.74	3.95	1.03	4.22	1.02
Other	1.63	2.88	2.32	3.38	2.60	3.34	2.18	1.23	2.25	1.23
Albumin	0.86	0.59	0.74	0.55	0.72	0.57	0.73	0.20	0.68	0.19
AIC	616.66		618.08		618.40		733.82		762.54	

* p<0.05;

** p<0.01

† HR: Hazard ratio;

‡ SE: Standard error

**Table 4:** Parametric regression model and Cox’s regression model fitted to pediatric patients’ data

covariate	Log-normal			Log-logistic			Weibull			Exponential			Cox’s model	
	HR^†^	SE^‡^		HR	SE		HR	SE		HR	SE		HR	SE
Weight														
<mean	1			1			1			1			1	
>mean	2.68**	0.98		6.60**	1.06		4.45**	1.16		4.96**	0.46		4.29**	0.46
Albumin	0.71	0.55		0.56	0.56		0.68	0.58		0.68	0.25		0.66	0.25
Child’s class														
A	1			1			1			1			1	
B	2.12*	1.17		3.86*	1.2		2.85*	1.31		2.63*	0.55		2.94*	0.54
C	1.02	1.63		1.08	1.71		1.14	1.81		1.02	0.78		1.14	0.78
AIC	260.89			263.40			267.37			307.88			298.10	

* p<0.05;

** p<0.01

† HR: Hazard ratio;

‡ SE: Standard error

## DISCUSSION

Liver transplantation is the treatment of choice for patients with ESLD. In this study, we investigated the effective factors on adult and pediatric patients’ survival after liver transplantation and compared Cox’s regression model with parametric models using AIC criteria.

According to the results, men needed liver transplantation more frequently than women. In similar studies, most of the patients in need of the procedure were males—the frequency was 61.2% in USA, 76.5% in Spain, and 52% in Brazil [21-23].

Hepatitis B in adults and biliary atresia in pediatric cases were the most common initial diagnosis of the liver disease. In most other studies on children, biliary duct atresia and hepatitis C and alcoholic cirrhosis in adults were the most common initial diagnoses [24, 25].

The mean MELD score in adults was higher, compared to similar studies conducted in the USA and Spain that reported a mean score of 16.1 and 16.2, respectively. However the mean PELD score for pediatric patients in our study was lower than that reported by other studies [22, 23, 26]. This would reflect better conditions of pediatric cases for the liver transplantation in our center.

The results of Cox’s regression and parametric models were fitted on adults in one direction and age was the only factor that predicted the adults’ survival after liver transplantation. Patients who were older than the mean age had lower survival than those below the mean age. In similar studies, higher age was associated with higher mortality after liver transplantation [27]. Patients aged 50–54 years had better survival than other studied age groups (55–59, 60–64, 65–69, ≥70 years old) (HR=1.06, p<0.05; HR=1.21, p<0.001; HR=1.42, p<0.001; HR=1.72, p<0.001, respectively) [28].

Weight and Child class were identified the independent prognostic factors affecting the pediatric cases’ survival after liver transplantation in both Cox’s regression and parametric models. Similar studies have shown that weight is an important factor affecting children patients’ survival (HR=4.6, p<0.05). Furthermore, pediatric patients weighing >2 SD below the mean had lower survival rates (RR=1.5, 95% CI: 0.95–2.40) [6, 29]. The results of this study showed that children’s weight less than the average at transplantation had a significantly lower survival rate than those who were above the average weight. According to several studies, children weighing less than 10 kg are considered high risk group and more likely to develop vascular and biliary complications and infections after transplantation. Moreover, weight gain in pediatric cases may lead to better results after liver transplantation [30-32].

The children in Child class A had better survival than those in classes B and C. In this study, the difference between the children in class A and B was significant; for low number of patients with class C disease, this difference was not significant. In similar studies, Child class was found an independent predictor in chronic liver failure after liver transplantation; survival of pediatric patients with class A disease was better than those in classes B and C. Survival of patients with class C disease was poorer (p<0.01) than that of patients with class B [33]. 

Although the hazard ratio of the two models is approximately similar, the AIC values reported for each model showed that parametric models had a better fit and were more powerful than Cox’s regression model. It was also found that among the parametric models, log-normal model with the lowest AIC value did better than others.

Some studies that compared the Cox’s regression model and parametric models have shown that the latter resulted in a better fit than the former model [34]. In many analyzing survival data that the proportionality assumption of Cox’s regression model does not satisfy, the log-normal parametric model is the model of choice.(35-37) Also, a simulation study showed that whether PH assumption is met or not, the log-logistic model is the best fitted model [35].

In conclusion, Cox’s regression is a well-known model applied in the analysis of survival data. Studies have indicated that under certain situations when the shape of the survival time is determined, the parametric models are more powerful and efficient than Cox’s regression model [9, 10, 20]. If the only basic assumption of this model (proportional hazards) is not met, parametric models are suitable alternative models to be used instead of Cox’s regression analysis.
